# (*E*)-1-(Pyridin-2-yl)-3-(3,4,5-trimeth­oxy­phen­yl)prop-2-en-1-one

**DOI:** 10.1107/S1600536811033198

**Published:** 2011-08-27

**Authors:** Hoong-Kun Fun, Thitipone Suwunwong, Suchada Chantrapromma

**Affiliations:** a X-ray Crystallography Unit, School of Physics, Universiti Sains Malaysia, 11800 USM, Penang, Malaysia; b Crystal Materials Research Unit, Department of Chemistry, Faculty of Science, Prince of Songkla University, Hat-Yai, Songkhla 90112, Thailand

## Abstract

In the title heteroaryl chalcone derivative, C_17_H_17_NO_4_, the dihedral angle between the pyridine and benzene rings is 10.82 (5)°. The two meth­oxy groups at the *meta* positions are essentially coplanar with the attached benzene rings [C—O—C—C torsion angles = −0.97 (14) and 179.47 (9)°], whereas the meth­oxy group at the *para* position is twisted from the attached ring with a C—O—C—C torsion angle of −104.48 (11)°. A C—H⋯O close contact involving two of the meth­oxy groups generates an *S*(6) ring motif. In the crystal, mol­ecules are linked by weak C—H⋯O inter­actions into columns along the *b* axis.

## Related literature

For background and applications of chalcones, see: Gacche *et al.* (2008 ▶); Isomoto *et al.* (2005 ▶); Jung *et al.* (2008 ▶); Nowakowska *et al.* (2001 ▶); Patil & Dharmaprakash (2008 ▶); Shibata (1994 ▶); Tewtrakul *et al.* (2003 ▶). For standard bond-length data, see: Allen *et al.* (1987 ▶). For hydrogen-bond motifs, see: Bernstein *et al.* (1995 ▶). For related structures, see: Fun *et al.* (2010 ▶); Suwunwong, Chantrapromma & Fun (2009 ▶); Suwunwong, Chantrapromma, Pakdeevanich & Fun (2009 ▶). For the stability of the temperature controller used in the data collection, see Cosier & Glazer, (1986 ▶).
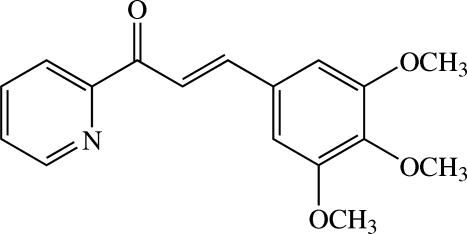


## Experimental

### 

#### Crystal data

C_17_H_17_NO_4_
*M* *_r_* = 299.32
Orthorhombic, 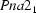

*a* = 25.0498 (5) Å
*b* = 3.9799 (1) Å
*c* = 14.3267 (3) Å
*V* = 1428.31 (5) Å^3^
*Z* = 4
Mo *K*α radiation
μ = 0.10 mm^−1^
*T* = 100 K
0.58 × 0.41 × 0.32 mm

#### Data collection

Bruker APEXII CCD area-detector diffractometer
Absorption correction: multi-scan (*SADABS*; Bruker, 2005 ▶) *T*_min_ = 0.945, *T*_max_ = 0.969
26700 measured reflections
3245 independent reflections
3130 reflections with *I* > 2σ(*I*)
*R*_int_ = 0.026

#### Refinement

*R*[*F* ^2^ > 2σ(*F* ^2^)] = 0.033
*wR*(*F* ^2^) = 0.091
*S* = 1.08
3245 reflections
202 parameters
1 restraint
H-atom parameters constrained
Δρ_max_ = 0.37 e Å^−3^
Δρ_min_ = −0.21 e Å^−3^

### 

Data collection: *APEX2* (Bruker, 2005 ▶); cell refinement: *SAINT* (Bruker, 2005 ▶); data reduction: *SAINT*; program(s) used to solve structure: *SHELXTL* (Sheldrick, 2008 ▶); program(s) used to refine structure: *SHELXTL*; molecular graphics: *SHELXTL*; software used to prepare material for publication: *SHELXTL* and *PLATON* (Spek, 2009 ▶).

## Supplementary Material

Crystal structure: contains datablock(s) global, I. DOI: 10.1107/S1600536811033198/lh5312sup1.cif

Structure factors: contains datablock(s) I. DOI: 10.1107/S1600536811033198/lh5312Isup2.hkl

Supplementary material file. DOI: 10.1107/S1600536811033198/lh5312Isup3.cml

Additional supplementary materials:  crystallographic information; 3D view; checkCIF report

## Figures and Tables

**Table 1:** Hydrogen-bond geometry (Å, °)

*D*—H⋯*A*	*D*—H	H⋯*A*	*D*⋯*A*	*D*—H⋯*A*
C16—H16*B*⋯O3^i^	0.96	2.49	3.3358 (14)	147
C16—H16*C*⋯O4	0.96	2.57	3.0817 (15)	113

Symmetry code: (i) 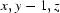.

**Fig. 1.**
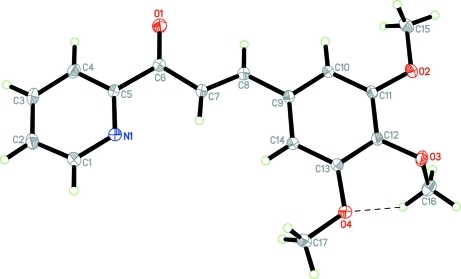
The molecular structure of the title compound, showing 50% probability displacement ellipsoids and the atom-numbering scheme. The dashed line indicates a weak intramolecular hydrogen bond.

**Fig. 2.**
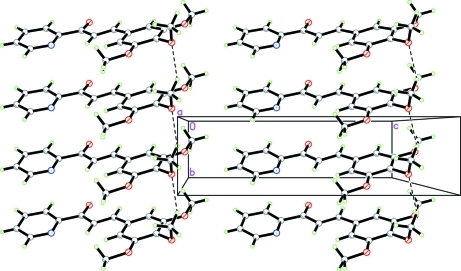
The crystal packing of the title compound, showing columns along the b axis. Hydrogen bonds are shown as dashed lines.

## supplementary crystallographic information

### Comment

Chalcones have a wide range of applications such as in non-linear optical devices (Patil & Dharmaprakash, 2008), electro-active fluorescent materials (Jung *et al.*, 2008), HIV-1 protease inhibitory (Tewtrakul *et al.*, 2003) as well as various biological properties including antioxidant (Gacche *et al.*, 2008), antibacterial (Nowakowska *et al.*, 2001; Isomoto *et al.*, 2005), and anticancer activities (Shibata, 1994). The title heteroaryl chalcone derivative (I) was synthesized during the course of our study on biological and pharmacological properties of synthetic chalcones and heteroaryl chalcones. Our result shows that (I) possesses moderate analgesic activity. It was also tested for antibacterial activity and found to be inactive.

The molecule of the title heteroaryl chalcone derivative (Fig. 1) exists in an *E* configuration with respect to the C7═C8 double bond [1.3456 (14) Å] and the torsion angle C6–C7–C8–C9 = -177.30 (10)°. The molecule is twisted as the dihedral angle between pyridine and 3,4,5-trimethoxyphenyl rings is 10.82 (5)°. The propenone bridge (C6-C8/O1) is nearly planar with the torsion angle O1–C6–C7–C8 = -7.16 (18)°. The mean plane through this bridge makes dihedral angles of 10.37 (8)° and 3.63 (8)° with the planes of pyridine and benzene rings, respectively. The three methoxy substituents of 3,4,5-trimethoxyphenyl unit have two different orientations in which the two methoxy groups at the *meta*-positions are co-planar with torsion angles C15–O2–C11–C10 = -0.97 (14)° and C17–O4–C13–C12 = 179.47 (9)°, whereas the third group at the *para*-position is twisted out of plane with a torsion angle of C16–O3–C12–C11 = -104.48 (11)°. A weak C16—H16C···O4 intramolecular interaction generates S(6) ring motif (Bernstein *et al.*, 1995) (Table 1). The bond angles are of normal values (Allen *et al.*, 1987) and are comparable with the related structures (Fun *et al.*, 2010; Suwunwong, Chantrapromma & Fun, 2009; Suwunwong, Chantrapromma, Pakdeevanich & Fun, 2009).

In the crystal (Fig. 2), the molecules are linked by weak intemolecular C16—H16B···O3^i^ interactions (Table 1) into columns along the *b* axis.

### Experimental

The title compound was synthesized by the condensation of 3,4,5-trimethoxybenzaldehyde (0.40 g, 2 mmol) with 2-acetylpyridine (0.20 g, 2 mmol) in ethanol (30 ml) in the presence of 30% NaOH(aq) (5 ml). After stirring in ice bath at 278 K for 3 h, the resulting pale yellow solid appeared and was then collected by filtration, washed with distilled water, dried and purified by repeated recrystallization from acetone. Pale yellow block-shaped single crystals of the title compound suitable for *x*-ray structure determination were recrystalized from acetone/ethanol (1:1 v/v) by the slow evaporation of the solvent at room temperature after four days, Mp. 434-435 K.

### Refinement

All H atoms were placed in calculated positions, with C-H = 0.93 Å, *U*_iso_ = 1.2*U*_eq_(C) for aromatic and CH and C-H = 0.96 Å, *U*_iso_ = 1.5*U*_eq_(C) for CH_3_ atoms. A rotating group model was used for the methyl groups. The highest residual electron density peak is located at 0.12 Å from C4 and the deepest hole is located at 0.28 Å from N1. A total of 2730 Friedel pairs were merged before final refinement as there is no significant anomalous dispersion for the determination of the absolute structure.

### Crystal data

**Table:**

C_17_H_17_NO_4_	*D*_x_ = 1.392 Mg m^−^^3^
*M**_r_* = 299.32	Melting point = 434–435 K
Orthorhombic, *P**n**a*2_1_	Mo *K*α radiation, λ = 0.71073 Å
Hall symbol: P 2c -2n	Cell parameters from 3245 reflections
*a* = 25.0498 (5) Å	θ = 2.2–35.0°
*b* = 3.9799 (1) Å	µ = 0.10 mm^−^^1^
*c* = 14.3267 (3) Å	*T* = 100 K
*V* = 1428.31 (5) Å^3^	Block, pale yellow
*Z* = 4	0.58 × 0.41 × 0.32 mm
*F*(000) = 632	

### Data collection

**Table:**

Bruker APEXII CCD area-detector diffractometer	3245 independent reflections
Radiation source: sealed tube	3130 reflections with *I* > 2σ(*I*)
graphite	*R*_int_ = 0.026
φ and ω scans	θ_max_ = 35.0°, θ_min_ = 2.2°
Absorption correction: multi-scan (*SADABS*; Bruker, 2005)	*h* = −34→40
*T*_min_ = 0.945, *T*_max_ = 0.969	*k* = −6→6
26700 measured reflections	*l* = −23→20

### Refinement

**Table:**

Refinement on *F*^2^	Primary atom site location: structure-invariant direct methods
Least-squares matrix: full	Secondary atom site location: difference Fourier map
*R*[*F*^2^ > 2σ(*F*^2^)] = 0.033	Hydrogen site location: inferred from neighbouring sites
*wR*(*F*^2^) = 0.091	H-atom parameters constrained
*S* = 1.08	*w* = 1/[σ^2^(*F*_o_^2^) + (0.0634*P*)^2^ + 0.087*P*] where *P* = (*F*_o_^2^ + 2*F*_c_^2^)/3
3245 reflections	(Δ/σ)_max_ = 0.001
202 parameters	Δρ_max_ = 0.37 e Å^−^^3^
1 restraint	Δρ_min_ = −0.21 e Å^−^^3^

### Special details

**Table:**

Experimental. The crystal was placed in the cold stream of an Oxford Cryosystems Cobra open-flow nitrogen cryostat (Cosier & Glazer, 1986) operating at 120.0 (1) K.
Geometry. All esds (except the esd in the dihedral angle between two l.s. planes) are estimated using the full covariance matrix. The cell esds are taken into account individually in the estimation of esds in distances, angles and torsion angles; correlations between esds in cell parameters are only used when they are defined by crystal symmetry. An approximate (isotropic) treatment of cell esds is used for estimating esds involving l.s. planes.
Refinement. Refinement of F^2^ against ALL reflections. The weighted R-factor wR and goodness of fit S are based on F^2^, conventional R-factors R are based on F, with F set to zero for negative F^2^. The threshold expression of F^2^ > 2sigma(F^2^) is used only for calculating R-factors(gt) etc. and is not relevant to the choice of reflections for refinement. R-factors based on F^2^ are statistically about twice as large as those based on F, and R- factors based on ALL data will be even larger.

### Fractional atomic coordinates and isotropic or equivalent isotropic displacement parameters (Å^2^)

**Table:**

	*x*	*y*	*z*	*U*_iso_*/*U*_eq_	
O1	0.24601 (4)	−0.1060 (3)	0.05761 (7)	0.02596 (19)	
O2	0.08957 (3)	0.0024 (2)	0.50009 (5)	0.01754 (15)	
O3	0.00155 (3)	0.3315 (2)	0.44825 (6)	0.01804 (15)	
O4	−0.01556 (3)	0.5094 (2)	0.26892 (6)	0.01841 (15)	
C4	0.23610 (4)	−0.0027 (3)	−0.13617 (8)	0.01727 (17)	
H4A	0.2657	−0.1252	−0.1167	0.021*	
C3	0.22725 (5)	0.0591 (3)	−0.23046 (8)	0.01872 (18)	
H3A	0.2504	−0.0255	−0.2754	0.022*	
C2	0.18322 (5)	0.2495 (3)	−0.25613 (7)	0.01913 (18)	
H2A	0.1766	0.2978	−0.3186	0.023*	
C1	0.14921 (4)	0.3668 (3)	−0.18655 (8)	0.01865 (19)	
H1A	0.1198	0.4947	−0.2042	0.022*	
N1	0.15656 (4)	0.3053 (2)	−0.09560 (6)	0.01632 (16)	
C5	0.19958 (4)	0.1232 (2)	−0.07164 (7)	0.01393 (16)	
C6	0.20623 (4)	0.0461 (3)	0.03063 (7)	0.01630 (17)	
C7	0.16244 (4)	0.1520 (3)	0.09225 (7)	0.01565 (17)	
H7A	0.1354	0.2880	0.0687	0.019*	
C8	0.16076 (4)	0.0553 (3)	0.18220 (7)	0.01548 (16)	
H8A	0.1893	−0.0724	0.2037	0.019*	
C9	0.11859 (4)	0.1310 (3)	0.24945 (7)	0.01371 (16)	
C10	0.12577 (4)	0.0284 (3)	0.34211 (7)	0.01412 (16)	
H10A	0.1570	−0.0816	0.3596	0.017*	
C11	0.08615 (4)	0.0913 (2)	0.40822 (6)	0.01337 (16)	
C12	0.03898 (4)	0.2565 (3)	0.38196 (7)	0.01385 (15)	
C13	0.03186 (4)	0.3557 (2)	0.28851 (7)	0.01376 (16)	
C14	0.07153 (4)	0.2953 (3)	0.22275 (7)	0.01422 (16)	
H14A	0.0669	0.3638	0.1612	0.017*	
C15	0.13684 (5)	−0.1702 (3)	0.52820 (8)	0.01924 (18)	
H15A	0.1339	−0.2324	0.5927	0.029*	
H15B	0.1672	−0.0258	0.5199	0.029*	
H15C	0.1412	−0.3686	0.4909	0.029*	
C16	−0.04469 (5)	0.1175 (3)	0.44731 (9)	0.02059 (19)	
H16A	−0.0727	0.2196	0.4831	0.031*	
H16B	−0.0357	−0.0964	0.4740	0.031*	
H16C	−0.0565	0.0864	0.3841	0.031*	
C17	−0.02386 (5)	0.6161 (3)	0.17457 (8)	0.01905 (19)	
H17A	−0.0580	0.7245	0.1695	0.029*	
H17B	−0.0229	0.4244	0.1339	0.029*	
H17C	0.0038	0.7709	0.1570	0.029*	

### Atomic displacement parameters (Å^2^)

**Table:**

	*U*^11^	*U*^22^	*U*^33^	*U*^12^	*U*^13^	*U*^23^
O1	0.0183 (4)	0.0404 (5)	0.0192 (4)	0.0113 (3)	0.0019 (3)	0.0060 (4)
O2	0.0191 (4)	0.0228 (4)	0.0107 (3)	0.0004 (3)	−0.0005 (2)	0.0022 (2)
O3	0.0168 (3)	0.0225 (3)	0.0148 (3)	−0.0011 (3)	0.0040 (3)	−0.0044 (3)
O4	0.0152 (3)	0.0256 (4)	0.0144 (3)	0.0049 (3)	0.0000 (3)	0.0018 (3)
C4	0.0158 (4)	0.0190 (4)	0.0171 (4)	0.0019 (3)	0.0036 (3)	0.0001 (3)
C3	0.0215 (5)	0.0190 (4)	0.0157 (4)	−0.0015 (3)	0.0065 (4)	−0.0015 (3)
C2	0.0220 (5)	0.0218 (4)	0.0136 (4)	−0.0027 (4)	0.0009 (3)	0.0001 (3)
C1	0.0178 (4)	0.0238 (5)	0.0144 (4)	0.0007 (3)	−0.0011 (3)	0.0019 (3)
N1	0.0134 (3)	0.0218 (4)	0.0137 (3)	0.0011 (3)	0.0000 (3)	0.0012 (3)
C5	0.0126 (4)	0.0168 (4)	0.0124 (3)	−0.0001 (3)	0.0017 (3)	0.0006 (3)
C6	0.0136 (4)	0.0210 (4)	0.0143 (4)	0.0013 (3)	0.0012 (3)	0.0012 (3)
C7	0.0140 (4)	0.0203 (4)	0.0126 (3)	0.0018 (3)	0.0013 (3)	0.0003 (3)
C8	0.0146 (4)	0.0188 (4)	0.0131 (4)	0.0012 (3)	0.0006 (3)	0.0003 (3)
C9	0.0132 (4)	0.0162 (4)	0.0117 (3)	−0.0009 (3)	0.0002 (3)	0.0000 (3)
C10	0.0133 (4)	0.0178 (4)	0.0112 (3)	0.0003 (3)	−0.0006 (3)	0.0003 (3)
C11	0.0149 (4)	0.0149 (4)	0.0103 (3)	−0.0014 (3)	−0.0004 (3)	−0.0001 (3)
C12	0.0144 (4)	0.0157 (4)	0.0114 (3)	−0.0005 (3)	0.0003 (3)	−0.0014 (3)
C13	0.0129 (4)	0.0157 (4)	0.0127 (3)	0.0003 (3)	−0.0005 (3)	−0.0011 (3)
C14	0.0130 (4)	0.0179 (4)	0.0118 (3)	−0.0001 (3)	−0.0001 (3)	0.0003 (3)
C15	0.0227 (5)	0.0193 (4)	0.0157 (4)	−0.0006 (3)	−0.0038 (4)	0.0031 (3)
C16	0.0180 (4)	0.0202 (4)	0.0235 (5)	−0.0009 (3)	0.0060 (4)	0.0008 (4)
C17	0.0177 (4)	0.0226 (5)	0.0168 (4)	0.0018 (3)	−0.0027 (3)	0.0038 (3)

### Geometric parameters (Å, °)

**Table:**

O1—C6	1.2285 (13)	C7—H7A	0.9300
O2—C11	1.3656 (12)	C8—C9	1.4611 (14)
O2—C15	1.4269 (14)	C8—H8A	0.9300
O3—C12	1.3676 (12)	C9—C10	1.4005 (14)
O3—C16	1.4376 (14)	C9—C14	1.4013 (14)
O4—C13	1.3654 (13)	C10—C11	1.3946 (14)
O4—C17	1.4320 (14)	C10—H10A	0.9300
C4—C3	1.3909 (16)	C11—C12	1.4034 (14)
C4—C5	1.3938 (14)	C12—C13	1.4072 (13)
C4—H4A	0.9300	C13—C14	1.3904 (14)
C3—C2	1.3877 (17)	C14—H14A	0.9300
C3—H3A	0.9300	C15—H15A	0.9600
C2—C1	1.3918 (16)	C15—H15B	0.9600
C2—H2A	0.9300	C15—H15C	0.9600
C1—N1	1.3386 (14)	C16—H16A	0.9600
C1—H1A	0.9300	C16—H16B	0.9600
N1—C5	1.3434 (13)	C16—H16C	0.9600
C5—C6	1.5061 (14)	C17—H17A	0.9600
C6—C7	1.4697 (14)	C17—H17B	0.9600
C7—C8	1.3456 (14)	C17—H17C	0.9600
			
C11—O2—C15	116.68 (9)	C11—C10—H10A	120.0
C12—O3—C16	114.63 (8)	C9—C10—H10A	120.0
C13—O4—C17	116.95 (8)	O2—C11—C10	124.28 (9)
C3—C4—C5	118.42 (10)	O2—C11—C12	115.64 (9)
C3—C4—H4A	120.8	C10—C11—C12	120.08 (9)
C5—C4—H4A	120.8	O3—C12—C11	119.55 (9)
C2—C3—C4	118.73 (9)	O3—C12—C13	120.83 (9)
C2—C3—H3A	120.6	C11—C12—C13	119.56 (9)
C4—C3—H3A	120.6	O4—C13—C14	124.05 (9)
C3—C2—C1	118.68 (10)	O4—C13—C12	115.58 (8)
C3—C2—H2A	120.7	C14—C13—C12	120.37 (9)
C1—C2—H2A	120.7	C13—C14—C9	119.80 (9)
N1—C1—C2	123.48 (10)	C13—C14—H14A	120.1
N1—C1—H1A	118.3	C9—C14—H14A	120.1
C2—C1—H1A	118.3	O2—C15—H15A	109.5
C1—N1—C5	117.24 (9)	O2—C15—H15B	109.5
N1—C5—C4	123.44 (9)	H15A—C15—H15B	109.5
N1—C5—C6	116.56 (8)	O2—C15—H15C	109.5
C4—C5—C6	119.96 (9)	H15A—C15—H15C	109.5
O1—C6—C7	123.89 (10)	H15B—C15—H15C	109.5
O1—C6—C5	119.75 (10)	O3—C16—H16A	109.5
C7—C6—C5	116.32 (9)	O3—C16—H16B	109.5
C8—C7—C6	121.12 (9)	H16A—C16—H16B	109.5
C8—C7—H7A	119.4	O3—C16—H16C	109.5
C6—C7—H7A	119.4	H16A—C16—H16C	109.5
C7—C8—C9	126.53 (9)	H16B—C16—H16C	109.5
C7—C8—H8A	116.7	O4—C17—H17A	109.5
C9—C8—H8A	116.7	O4—C17—H17B	109.5
C10—C9—C14	120.19 (9)	H17A—C17—H17B	109.5
C10—C9—C8	118.17 (9)	O4—C17—H17C	109.5
C14—C9—C8	121.63 (9)	H17A—C17—H17C	109.5
C11—C10—C9	120.00 (9)	H17B—C17—H17C	109.5
			
C5—C4—C3—C2	1.43 (16)	C15—O2—C11—C12	179.12 (9)
C4—C3—C2—C1	−0.96 (17)	C9—C10—C11—O2	−179.72 (9)
C3—C2—C1—N1	−0.13 (18)	C9—C10—C11—C12	0.18 (15)
C2—C1—N1—C5	0.71 (17)	C16—O3—C12—C11	−104.48 (11)
C1—N1—C5—C4	−0.19 (15)	C16—O3—C12—C13	78.28 (12)
C1—N1—C5—C6	−177.94 (10)	O2—C11—C12—O3	3.10 (14)
C3—C4—C5—N1	−0.88 (16)	C10—C11—C12—O3	−176.82 (9)
C3—C4—C5—C6	176.80 (10)	O2—C11—C12—C13	−179.63 (8)
N1—C5—C6—O1	−176.67 (11)	C10—C11—C12—C13	0.45 (14)
C4—C5—C6—O1	5.50 (16)	C17—O4—C13—C14	−0.67 (15)
N1—C5—C6—C7	5.36 (14)	C17—O4—C13—C12	179.47 (9)
C4—C5—C6—C7	−172.47 (10)	O3—C12—C13—O4	−3.86 (14)
O1—C6—C7—C8	−7.16 (18)	C11—C12—C13—O4	178.91 (9)
C5—C6—C7—C8	170.72 (10)	O3—C12—C13—C14	176.28 (9)
C6—C7—C8—C9	−177.30 (10)	C11—C12—C13—C14	−0.96 (14)
C7—C8—C9—C10	−175.49 (10)	O4—C13—C14—C9	−179.04 (9)
C7—C8—C9—C14	5.80 (17)	C12—C13—C14—C9	0.81 (14)
C14—C9—C10—C11	−0.33 (15)	C10—C9—C14—C13	−0.16 (15)
C8—C9—C10—C11	−179.06 (9)	C8—C9—C14—C13	178.52 (9)
C15—O2—C11—C10	−0.97 (14)		

### Hydrogen-bond geometry (Å, °)

**Table:**

*D*—H···*A*	*D*—H	H···*A*	*D*···*A*	*D*—H···*A*
C16—H16B···O3^i^	0.96	2.49	3.3358 (14)	147
C16—H16C···O4	0.96	2.57	3.0817 (15)	113

Symmetry codes: (i) *x*, *y*−1, *z*.
